# Mesenchymal Stem Cells of Epicardial Adipose Tissue Show Differences in Immunophenotype and Osteogenic Potential in Patients with Coronary and Non-Coronary Heart Disease

**DOI:** 10.3390/cells15141270

**Published:** 2026-07-15

**Authors:** Olga V. Gruzdeva, Tamara A. Slesareva, Evgeniya E. Gorbatovskaya, Sofia E. Dolmatova, Yulia A. Dyleva, Elena V. Fanaskova, Alexander N. Kokov, Asliddin B. Nishonov, Olga L. Barbarash

**Affiliations:** Federal State Budgetary Institution Research Institute for Complex Issues of Cardiovascular Diseases, 6, boulevard named after academician L.S. Barbarasha, Kemerovo 650002, Russia; o_gruzdeva@mail.ru (O.V.G.); eugenia.tarasowa@yandex.ru (E.E.G.); soniad_30_09@mail.ru (S.E.D.); dyleva87@yandex.ru (Y.A.D.); fanaev@kemcardio.ru (E.V.F.); dr.kokov@gmail.com (A.N.K.); aslidin_nishonov@mail.ru (A.B.N.); reception@kemcardio.ru (O.L.B.)

**Keywords:** mesenchymal stem cells, epicardial adipose tissue, coronary artery calcification, ischemic heart disease, aortic stenosis

## Abstract

Coronary artery calcification (CAC) is a pressing issue in cardiology. Mesenchymal stem cells (MSCs) derived from epicardial adipose tissue (EAT) may serve as a source of osteoblasts in the cardiovascular system. This study aimed to evaluate and compare the immunophenotype, proliferation, and osteogenic potential of EAT-MSCs from patients with coronary artery disease (CAD) and those with aortic stenosis (AS). The immunophenotype of MSCs was analyzed based on key markers: CD105, CD90, CD73, CD31, CD34, CD45, HLA-DR. Cell proliferation was assessed by the doubling time of the population and the specific growth rate of the cultures. The osteogenic potential was evaluated by the expression levels of the *RUNX2*, *SPP1*, *ALPL*, and *BGLAP* genes using PCR, as well as the protein levels in supernatants via ELISA. Qualitative detection of osteogenic proteins in cells by immunofluorescence staining. The intensity of extracellular matrix mineralization was measured using photometric methods. CD73 expression in EAT-MSCs from CAD patients was nearly 50% lower than in EAT-MSC cultures from AS patients. Following osteogenic induction, EAT-MSCs from CAD patients showed increased expression of osteogenic marker genes and their corresponding proteins. The intensity of extracellular matrix mineralization by osteoblasts derived from EAT-MSCs of CAD patients was higher than in the comparison group. EAT-MSCs from CAD patients with coronary calcification demonstrated reduced CD73 expression in culture and enhanced osteogenic potential compared to patients without coronary artery involvement.

## 1. Introduction

Coronary artery disease (CAD) remains the most common pathology of the cardiovascular system [1]. A significant complication that worsens the course of CAD is coronary artery calcification (CAC). According to most authors, in the age group over 70 years, CAC occurs in more than 90% of men and more than 67% of women [2]. CAC leads to decreased vascular elasticity, abnormal vasomotor responses, impaired myocardial perfusion, and, consequently, a worse prognosis and increased risk of adverse outcomes in CAD [3]. Furthermore, the presence of CAC complicates interventional coronary procedures aimed at myocardial revascularization and is associated with a higher risk of intraoperative complications and long-term cardiovascular events [4]. Recently, vascular calcification has been viewed as an active, cell-mediated process similar to osteogenesis. It is believed that cells within the arterial wall reprogram their genetic expression patterns, transform into osteoblast-like cells, and initiate extracellular matrix mineralization [5]. Various authors have suggested that pericytes, adventitial stem cells, or vascular smooth muscle cells (VSMCs) are involved [6]. However, little attention has been paid to the role of MSCs from adipose tissue surrounding the heart and vessels.

Epicardial adipose tissue (EAT) is a local fat depot directly adjacent to the heart and coronary vessels. Assessment of EAT volume is particularly important for cardiovascular risk stratification. To date, it has been shown that EAT volume is independently associated with the degree of coronary calcification [7]. The involvement of EAT in the pathogenesis of CAC is further supported by studies demonstrating the osteogenic activity of this fat depot. For example, M. Luna et al. found high expression of genes encoding bone morphogenetic protein 2 and osteopontin in the EAT of patients with coronary calcification compared to patients without vascular calcification [8]. Although the cellular source of osteogenic marker expression in EAT remains insufficiently studied, there is reason to believe that this activity may be mediated by MSCs present in the stromal-vascular fraction of this fat depot, which possess osteogenic differentiation potential [9]. The theory that osteogenic differentiation of EAT-MSCs underlies the pathogenesis of CAC is one of the least explored topics in the current literature. To test this hypothesis, we studied the immunophenotype, proliferation, and osteogenesis of EAT-MSCs from patients with calcifying coronary artery disease and from patients with valvular heart disease without signs of vascular calcification.

## 2. Materials and Methods

In this research study, two patient groups were formed: one with coronarogenic pathology (CAD) and one with non-coronarogenic heart pathology (aortic valve stenosis (AS)). Each group included 30 patients with the respective nosological form of cardiovascular disease. Written informed consent was obtained from all study participants. Inclusion criteria for the main patient group were: established diagnosis of CAD, indications for open coronary artery bypass grafting (based on coronary angiography (CAG) data), absence of aortic valve disease, and age under 75 years. Inclusion criteria for the comparison group were: established diagnosis of aortic stenosis, indications for open aortic valve replacement, absence of atherosclerotic coronary artery lesions according to CAG, and age under 75 years. Exclusion criteria were: patient age over 75 years, type 1 or type 2 diabetes mellitus (history or diagnosed during hospitalization), clinically significant comorbidities (renal or hepatic failure, anemia, infectious or inflammatory diseases during exacerbation, oncological or autoimmune diseases), and patient refusal to participate.

Clinical characteristics of the patients are presented in Table 1.

### 2.1. Assessment of Coronary Artery Calcification

All patients underwent ECG-gated multidetector computed tomography (MDCT) of the heart on a 64-slice spiral CT scanner (Siemens Somatom 64, Siemens, Erlangen, Germany) to assess the degree of coronary artery calcification. The acquired native images in DICOM format were post-processed on a multimodal Leonardo workstation (Siemens, Germany). The coronary artery calcium score was determined using the Agatston method with the Syngo Calcium Scoring software package (syngo.via) (Siemens AG Medical Solutions, Erlangen, Germany). The total calcium score (CaScore Total) was assessed semi-automatically. According to the Agatston classification, the following grades of coronary artery calcification were defined based on the total calcium score: CS = 0—no calcification; CS = 1–10—minimal calcification; CS = 11–100—mild calcification; CS = 101–400—moderate calcification; CS > 400—severe/extensive calcification.

### 2.2. Coronary Angiography

Coronary angiography was performed in all patients diagnosed with coronary artery disease (CAD) and aortic stenosis (AS). Coronary artery atherosclerosis was diagnosed using the technique described by M.P. Judkins (1967) on an Innova angiography system (USA) [10]. First, puncture of the femoral or radial artery was performed according to the technique described by Seldinger (1952). Xenetic-350 was used as a radiopaque contrast agent. Lesions of the coronary bed were assessed according to the presence of single-, double-, or triple-vessel disease. In addition, quantitative assessment was performed using the SYNTAX Score scoring system via an online calculator (http://www.syntaxscore.com/, accessed on 20 March 2025) to evaluate anatomically complex coronary lesions, including vessel tortuosity, bifurcation involvement, calcification, thrombosis, presence and length of stenosis, occlusion, its duration, and morphology.

### 2.3. Laboratory Methods

Serum levels of total cholesterol (TC), high-density lipoprotein cholesterol (HDL-C), low-density lipoprotein cholesterol (LDL-C), very low-density lipoprotein cholesterol (VLDL-C), triacylglycerols (TAG), lipoprotein(a), and C-reactive protein (CRP) were measured using an automatic biochemical analyzer (Cobas c 311, Switzerland) with standard test systems from Roche Diagnostics. Human interleukin-6 (IL-6) levels were determined in serum using an enzyme-linked immunosorbent assay (ELISA) kit (FineTest, Anyan, China).

### 2.4. Isolation and Cultivation of EAT-MSCs

To obtain MSC cultures, tissue samples (epicardial adipose tissue) were collected during open heart surgery (coronary artery bypass grafting or valve repair). The EAT source was the left heart region. Adipose tissue biopsies were thoroughly washed with sterile phosphate-buffered saline (PBS) (Gibco, Grand Island, NY, USA) supplemented with penicillin (600 U/mL) (Gibco, Grand Island, NY, USA) and streptomycin (300 mg/mL, Gibco, Grand Island, NY, USA) to clean the tissue surface of clots, erythrocytes, and local anesthetics. The EAT was then transferred to a culture dish and cut into small pieces. Fragmented EAT was placed into 25 cm^2^ culture flasks (Biologix, Jinnan, China). The tissue was incubated in a CO_2_ incubator (5% CO_2_, 95% air, 37 °C) in MSC growth medium (MesenCult Proliferation Kit, STEMCELL Technologies, Vancouver, BC, Canada) supplemented with antibiotics and antimycotics (100 U/mL penicillin, 100 U/mL streptomycin, 0.4% amphotericin B, Gibco, Grand Island, NY, USA). The culture medium was changed every 3–4 days. When primary cells reached 80–90% confluence, they were treated with 0.25% trypsin containing 0.02% EDTA (Trypsin/EDTA, STEMCELL Technologies, Vancouver, BC, Canada) and transferred to 75 cm^2^ culture flasks (Biologix, Jinan, China) for passaging up to passage 3. Cells at passage 3 were used for experiments.

### 2.5. Flow Cytometry

Surface marker expression was assessed in EAT-MSCs at passage 3. A total of 30 MSC cultures derived from EAT of CAD patients and 30 cultures derived from EAT of AS patients were analyzed (one technical replicate per culture). The following panel of directly conjugated monoclonal antibodies and the corresponding isotype controls was used for flow cytometric analysis: CD90 FITC (BC, IM1839U), CD73 APC-Cy7 (BioLegend, 344022, San Diego, CA, USA), CD105 PE (BioLegend, 323206, San Diego, CA, USA), CD34 APC (BC, IM2472U), CD31 PE (BioLegend, 303106, San Diego, CA, USA), CD45 Pacific Blue (BioLegend, 304029, San Diego, CA, USA), and HLA-DR APC (BioLegend, 327022, San Diego, CA, USA). Antibodies were added to a suspension containing 25 × 10^4^ cells in the volume recommended by the manufacturer, followed by incubation at room temperature in the dark. Stained samples were resuspended in PBS and analyzed on a CytoFlex flow cytometer (Brea, CA, USA) using CytExpert 2.1 software. All samples were analyzed under identical instrument settings. 

### 2.6. Assessment of Proliferative Activity

Cell proliferation was analyzed using the CCK-8 kit (Servicebio, Wuhan, China). A total of 30 EAT-MSC cultures from CAD patients and 30 cultures from AS patients were examined (number of technical replicates: 2). To generate a calibration curve, a known number of passage 3 cells (1 × 10^4^) was serially diluted in culture medium to a concentration of 1 × 10^3^. Known cell numbers were transferred to 96-well culture plates pre-coated with 10 mg/mL fibronectin (PanEco, Moscow, Russia) at 100 µL per well. For the experiment, cells were seeded in duplicate at 1 × 10^3^ cells/well. After cell attachment, 10 µL of CCK-8 reagent was added to the standard samples. Following incubation for 2 h at 37 °C, optical density was read using a microplate reader (Erba LisaScan, Brno, Czech Republic) at 450 nm. Experimental samples were then tested similarly on days 3, 7, and 10 of culture.

Population doubling time was calculated for passage 3 cells using the formula: TD = log_2_ (2) × t/[log_2_(N_1_/N_0_)], where is the population growth time, N_1_ is the cell number after time t, and N_0_ is the initial cell number. The specific growth rate was calculated as μ = (ln X_1_ − ln X_0_)/(T_1_ − T_0_).

### 2.7. β-Galactosidase Staining

A total of 30 EAT-MSC cultures from CAD patients and 30 cultures from AS patients were examined (number of replicates: 1). Passage 3 cells were seeded into 6-well plates (4.5 × 10^4^ cells/well) pre-coated with fibronectin (10 μg/mL) (PanEco, Moscow, Russia). After MSC attachment, the culture medium was removed, and the cells were stained using a β-galactosidase staining kit for senescent cells (Servicebio, Wuhan, China) according to the manufacturer’s instructions. Stained cells were counted and expressed as a proportion of the total number of cells per well at a ratio of 1:100.

### 2.8. Osteogenic Differentiation of EAT-MSCs

Passage 3 cells were used. Thirty EAT-MSC cultures from CAD patients and 30 from AS patients were examined (1 technical replicate each). Cell suspension was added to culture plates pre-coated with human fibronectin (10 μg/mL) (PanEco, Moscow, Russia): 6-well plates–4.5 × 10^4^ cells/well (for mRNA isolation and ELISA of culture supernatants), and 24-well plates–5 × 10^3^ cells/well (for Alizarin Red staining). Osteogenic differentiation was induced using ready-to-use MSCgo™ Osteogenic Differentiation Medium (Sartorius, Göttingen, Germany). Differentiation proceeded for 21 days, with medium changes every 3 days.

### 2.9. Assessment of Osteogenic Potential by Osteogenic Gene Expression Using PCR

Expression levels of target genes (RUNX2, SP7, BGLAP, ALPL, SPP1) were evaluated using quantitative real-time PCR (qPCR) on a ViiA 7 Real-Time PCR System (Applied Biosystems, Waltham, MA, USA). PCR was performed in 96-well plates containing analyzed samples, five standards with 2-fold dilutions (for standard curve generation and amplification efficiency analysis), and a negative control (reaction mix without cDNA). For each analyzed sample, 25 μL of reaction mix was prepared containing 12.5 μL of BioMaster UDG HS-qPCR Lo-ROX SYBR (2×) master mix (Biolabmix, Novosibirsk, Russia, Cat. No. Biolabmix-MHR033-2040), 0.125 μL each of forward and reverse SYBR Green primers (Eurogen, Moscow, Russia), and 12.25 μL of synthesized cDNA at 20 ng/μL. Each sample, standard, and negative control was analyzed in triplicate. The amplification protocol included three stages: anti-contamination treatment for 2 min at 50 °C (1 cycle), initial denaturation for 5 min at 95 °C (1 cycle), and 40 cycles of denaturation for 15 s at 95 °C, annealing for 15 s at 65 °C, and elongation for 30 s at 72 °C. Amplification quality was assessed by analyzing amplification curves and standard curves using QuantStudio™ Real-Time PCR Software v.1.3 (Applied Biosystems, Waltham, MA, USA). Amplification was considered successful with efficiency values of 90–110%, R^2^ ≥ 0.980, and no amplification in the negative control. PCR results were normalized relative to the geometric mean Ct value of three reference genes: ACTB (β-actin), GAPDH (glyceraldehyde-3-phosphate dehydrogenase), B2M (beta-2-microglobulin) (Eurogen JSC, Moscow, Russia). Table 2 lists the primers used in the study. The comparative Ct method (2^−ΔΔCt^ method by Livak) was used for data analysis. Cells were examined on days 3, 15, and 21 of differentiation.

### 2.10. Enzyme-Linked Immunosorbent Assay (ELISA) of EAT-MSC Culture Lysates

Cells were examined at passage 3 and on days 3, 15, and 21 of differentiation. Before analysis, cultures were lysed to release intranuclear and cytoplasmic proteins not secreted into the extracellular space. Each well was washed three times with 1× PBS and treated with RIPA (Strong) lysis buffer (Servicebio, Wuhan, China) at 250 μL/well for 5 min. Cells were then collected and incubated on ice for 30 min. The resulting lysate was centrifuged, and the supernatant containing proteins was separated from cell debris and frozen at −80 °C until analysis. Protein levels of ALP (tissue-nonspecific alkaline phosphatase), RUNX2, OPN (osteopontin, encoded by SPP1), and OCN (osteocalcin, encoded by BGLAP) were measured using test systems: Alkaline Phosphatase ELISA Kit, Human RUNX2 ELISA Kit, Osteopontin Human ELISA Kit, and Human OC/BGP (Osteocalcin) ELISA Kit (FineTest, Anyan, China) according to the manufacturer’s protocols.

### 2.11. Immunofluorescence Staining

MSCs cultured in osteogenic medium were stained on day 15 of differentiation using the staining protocol recommended by the manufacturer. The following primary antibodies were used: anti-RUNX2 (ab23981, rabbit anti-human) at a 1:250 dilution, anti-osteopontin (ab8448, rabbit anti-human) at a 1:250 dilution, and anti-osteocalcin (ab13421, mouse anti-human) at a 1:250 dilution (Abcam, Cambridge (UK) ). As secondary antibodies, Donkey anti-rabbit preadsorbed (ab150061) and Donkey anti-mouse preadsorbed (ab150110) were used at a 1:500 dilution. Alkaline phosphatase was detected using directly conjugated monoclonal antibodies (ABIN7782561) at a 1:250 dilution. Cell nuclei were counterstained with the blue fluorescent nucleic acid dye DAPI (1:100 dilution). Fluorescence was visualized using a confocal microscope.

### 2.12. Alizarin Red Staining and Photometric Quantification of Staining Intensity

To confirm successful osteogenic differentiation, cultures were stained with Alizarin Red on day 21 of cultivation. Cells in 24-well plates were washed with PBS, and 500 μL of 4% paraformaldehyde was added for 30 min. After washing with PBS, 500 μL of 2% Alizarin Red solution (Lenreactive, Saint Petersburg, Russia) was added for 1 h in the dark. Each well was then washed once with 1 mL PBS, and results were documented by micro- and macrophotography. For photometric determination of staining intensity, 250 μL of 10% acetic acid (Lenreactive, Saint Petersburg, Russia) was added to each well of the 24-well plate with stained cells. The supernatant was collected into Eppendorf tubes, 75 μL of 10% ammonium hydroxide (Alfa Aesar, Ward Hill, MA, USA) was added, and 100 μL of the resulting solution was transferred to a 96-well plate for optical density measurement using a microplate reader (Erba LisaScan, Brno, Czech Republic) at 450 nm. To determine the concentration of calcium-bound Alizarin Red, a calibration curve was generated by measuring the optical density of Alizarin Red solutions at concentrations ranging from 200 to 3.125 μmol/L.

### 2.13. Statistical Analysis

Data were collected in a database using Microsoft Excel (Microsoft Corporation, Richmond, WA, USA). Statistical processing and graphical presentation were performed using IBM SPSS Statistics 27 (IBM, New York, NY, USA) and GraphPad Prism 8.0 (Dotmatics, Woburn, MA, USA). Data are presented as median and 25th and 75th percentiles: Me (Q25; Q75). For comparisons of quantitative variables between two independent groups with non-normal distribution, the nonparametric Mann–Whitney U test was used. The critical significance level was *p* < 0.05. Comparisons between three related groups were performed using the Friedman test, followed by Bonferroni adjustment for multiple comparisons. A *p*-value < 0.016 was considered statistically significant. Kendall’s tau correlation coefficient was applied to evaluate the relationship between two quantitative variables when the data deviated from normality in small samples (n ≤ 30).

## 3. Results

### 3.1. Comparative Assessment of the Immunophenotype of EAT-MSCs from CAD and AS Patients

At passage 3, the proportion of CD105-positive cells in EAT-MSC cultures from CAD patients was significantly higher than in EAT-MSC cultures from AS patients (*p* = 0.043). Furthermore, EAT-MSC cultures from patients with coronary artery involvement consisted of only 50% CD73-positive cells, which was 1.5-fold lower than the proportion of CD73-positive cells in EAT-MSC cultures from patients with aortic valve disease (*p* = 0.0012). The proportion of CD90-positive cells did not differ according to nosology. Cells from both groups showed almost no expression of the surface markers CD45, CD34, CD31, or HLA-DR (Table 3).

### 3.2. Comparative Assessment of the Proliferative Potential of EAT-MSCs from CAD and AS Patients

**Figure 1:**
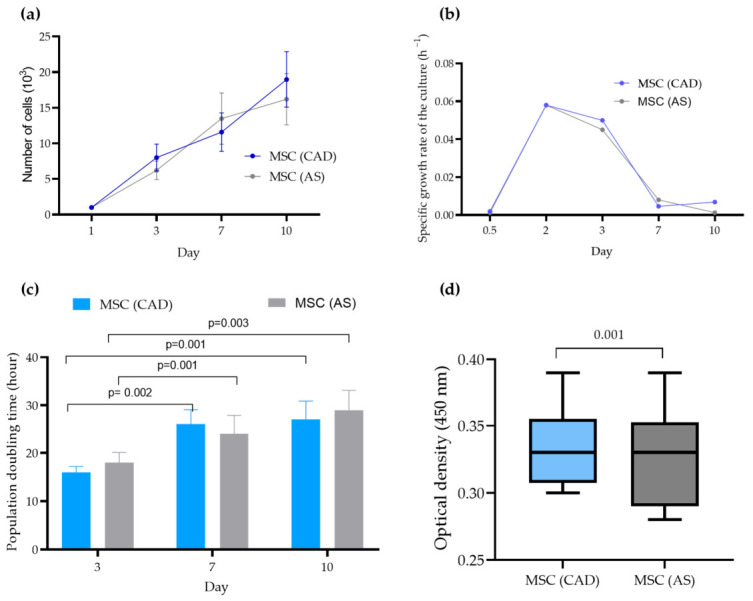
Assessment of growth parameters of EAT-MSC cultures from CAD and AS patients. Note: (**a**) Proliferation curves on days 3, 7, and 10; (**b**) Specific growth rate of a crop (**c**) Population doubling time on days 3, 7, and 10; (**d**) Cell viability on day 3.

The number of cells in cultures from both groups increased gradually from day 1 to day 10, with no differences at any observation time point (Figure 1a). The specific growth rate of MSCs was close to zero 12 h after culture initiation and increased by day 2. On day 3, a slight decrease in cell division rate was observed compared to day 2. On day 7, a significant decrease in the specific division rate of EAT-MSCs was detected in both patient groups. This growth slowdown persisted on day 10 (Figure 1b). Population doubling time was shortest by day 3 and increased significantly by days 7 and 10, indicating slower cell division at these time points. This parameter, as well as cell viability on day 3, did not differ between cultures from CAD and AS patients (Figure 1c,d). Thus, EAT-MSC cultures from CAD and AS patients showed virtually no differences in growth parameters or cell viability.

### 3.3. Comparative Assessment of Cellular Senescence in EAT-MSC Cultures from CAD and AS Patients

**Figure 2:**
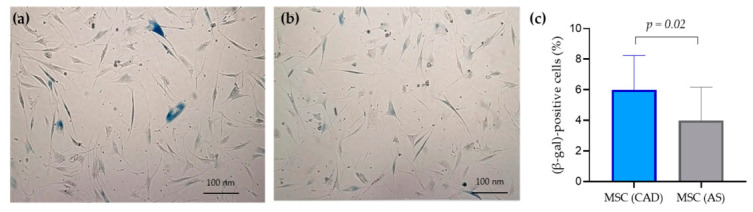
Micrographs of EAT MSCs stained with β-galactosidase at passage 3, (β-gal)-positive cells are stained blue: (**a**) EAT MSCs from patients with CAD; (**b**) EAT MSCs from patients with AS; (**c**) comparative assessment of the proportion of old cells in the cultures.

At passage 3, EAT-MSCs displayed signs of cellular senescence, as confirmed by the presence of β-galactosidase (β-gal)-positive cells with intense blue staining. However, in cultures derived from CAD patients, the proportion of β-gal-positive cells was 25% higher compared to cultures from AS patients (Figure 2).

### 3.4. Comparative Assessment of the Osteogenic Potential of EAT-MSCs from CAD and AS Patients

**Figure 3:**
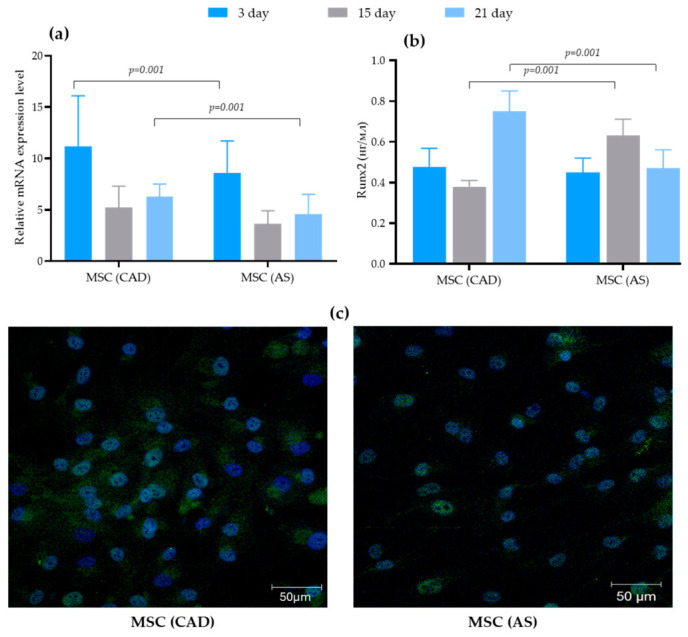
Detection of RUNX2 in EAT-MSC cultures from CAD and AS patients during osteogenic differentiation. Note: (**a**) Relative expression level of *RUNX2* on days 3, 15, and 21 of osteogenic differentiation; (**b**) RUNX2 protein levels in culture supernatants; (**c**) Immunofluorescence staining of RUNX2 protein in EAT-MSCs (intranuclear localization).

Osteogenic activity was assessed on days 3, 7, and 21 of EAT-MSC differentiation by qualitative and quantitative determination of osteogenic markers, including proteins and their corresponding encoding genes. The transcription factor *RUNX2* showed higher activity in MSCs from CAD patients on days 3 and 15 than in the AS group; however, by day 21, its expression levels were comparable between the two groups (Figure 3a). The concentration of the RUNX2 protein in supernatants changed non-linearly. On day 3, levels were equivalent in both cultures; on day 15, MSCs from AS patients more actively synthesized this protein; by day 21, the RUNX2 concentration in supernatants of MSCs from CAD patients significantly exceeded that in cultures from the AS group (Figure 3b).

**Figure 4:**
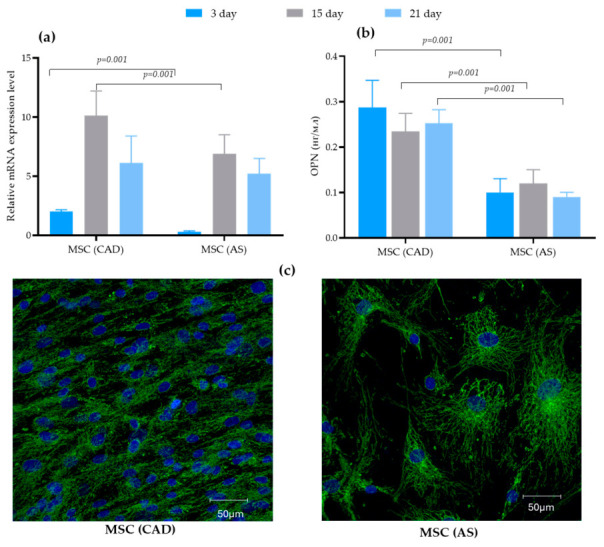
Detection of osteopontin in EAT-MSC cultures from CAD and AS patients during osteogenic differentiation. Note: (**a**) Relative expression level of the gene encoding osteopontin (*SPP1*) on days 3, 15, and 21 of osteogenic differentiation; (**b**) Osteopontin protein levels in culture supernatants; (**c**) Immunofluorescence staining of osteopontin protein in EAT-MSCs (intracellular localization).

Expression of the *SPP1* gene on days 3 and 15 of osteogenic differentiation was significantly higher in cultures derived from EAT of CAD patients than in MSC cultures from AS patients. By day 21, these differences disappeared, and both cultures showed almost identical gene activity (Figure 4a). In contrast, the level of osteopontin (the protein encoded by *SPP1*) was consistently higher in cultures from EAT of patients with coronary artery involvement than in the comparison group throughout the differentiation period (Figure 4b).

**Figure 5:**
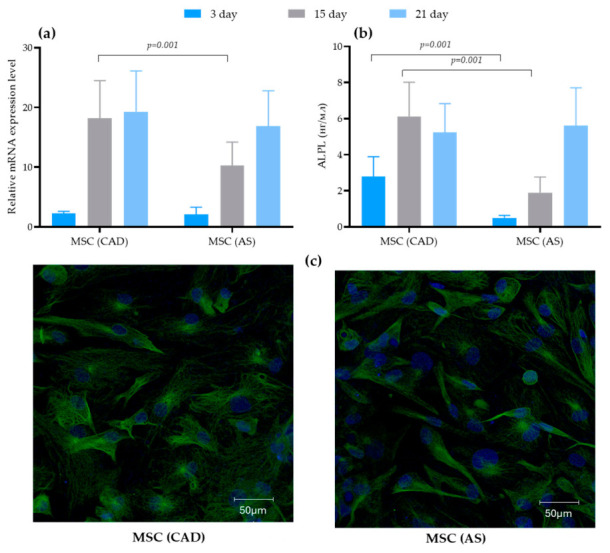
Detection of alkaline phosphatase (ALP) in EAT-MSC cultures from CAD and AS patients during osteogenic differentiation. Note: (**a**) Relative expression level of the gene encoding ALP (*ALPL*) on days 3, 15, and 21 of osteogenic differentiation; (**b**) ALP protein levels in culture supernatants; (**c**) Immunofluorescence staining of ALP protein in EAT-MSCs (intracellular localization).

The mRNA level of the gene encoding tissue-nonspecific alkaline phosphatase, *ALPL*, differed between the studied cultures only on day 15 of differentiation and was higher in EAT-MSCs from CAD patients (Figure 5a). Similarly, the concentration of alkaline phosphatase in the supernatants was significantly higher in these cultures on days 15 and 21 of differentiation (Figure 5b).

**Figure 6:**
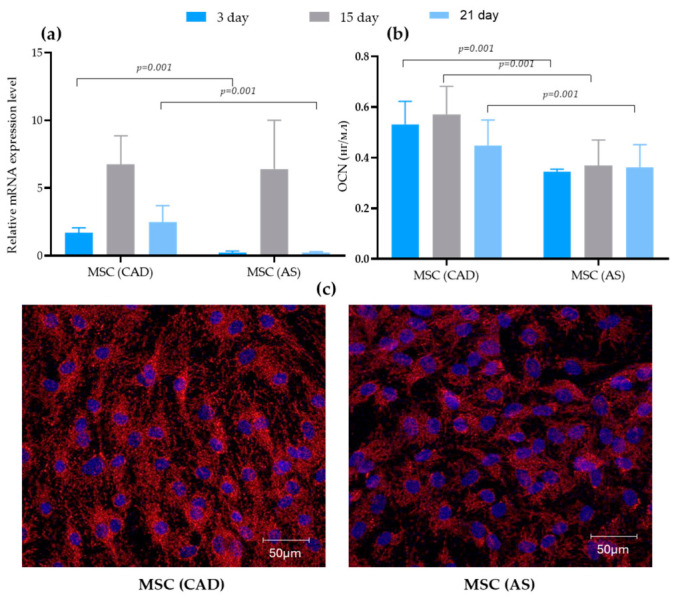
Detection of osteocalcin in EAT-MSC cultures from CAD and AS patients during osteogenic differentiation. Note: (**a**) Relative expression level of the gene encoding osteocalcin (*BGLAP*) on days 3, 15, and 21 of osteogenic differentiation; (**b**) Osteocalcin protein levels in culture supernatants; (**c**) Immunofluorescence staining of osteocalcin protein in EAT-MSCs (extracellular localization).

On day 3 of osteogenic differentiation, osteoblasts derived from EAT-MSCs of CAD patients showed an eight-fold increase in *BGLAP* gene expression compared to the same cells from AS patients (*p* = 0.001). On day 15, expression levels of this marker remained similar in both groups. By day 21, *BGLAP* mRNA levels decreased in both groups compared to day 15; however, in CAD patients, they remained significantly higher than in AS patients (Figure 6a). Osteocalcin protein levels in supernatants of EAT-MSCs from CAD patients were significantly higher than those from AS patients throughout the differentiation period. Interestingly, osteocalcin levels remained relatively stable within each group during differentiation, demonstrating consistency (Figure 6b).

Thus, analysis of genetic and molecular markers of osteogenic differentiation in MSCs from cardiac adipose tissue revealed that osteogenic activity is higher in cells from patients with atherosclerotic and calcifying coronary artery disease than in cells from patients without this pathology.

### 3.5. Comparative Assessment of Extracellular Matrix Mineralization Intensity by Osteoblasts Derived from EAT-MSCs of CAD and AS Patients

**Figure 7:**
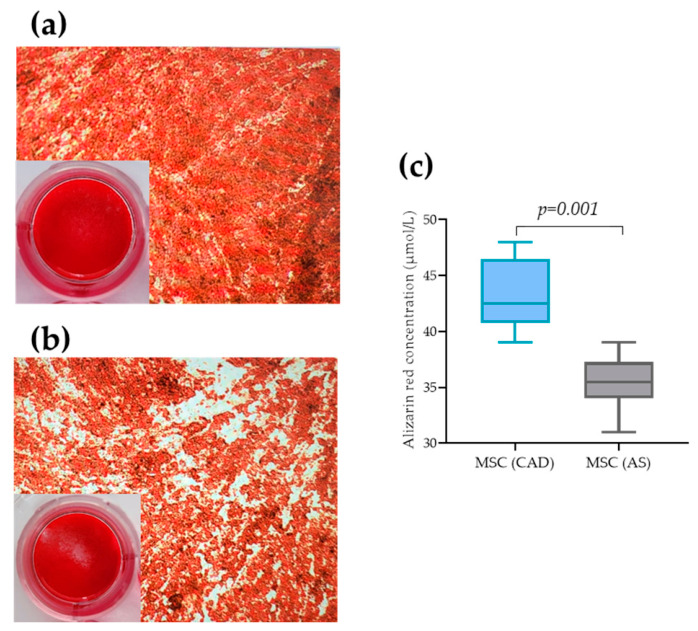
Micro- and macrophotographs of Alizarin Red staining of osteoblasts derived from EAT-MSCs of CAD and AS patients, and quantitative measurement of staining intensity. Note: (**a**) Osteoblasts from EAT-MSCs of CAD patients; (**b**) Osteoblasts from EAT-MSCs of AS patients; (**c**) Photometric measurement of calcium-bound Alizarin Red concentration.

Differentiated osteoblast cultures were stained with a dye with affinity for calcium salts to assess the calcification activity of the extracellular matrix. Visual evaluation showed that calcium in osteoblast cultures derived from EAT-MSCs of CAD patients was uniformly distributed across the cell monolayer, with more than 80% of the well bottom area covered by calcium (Figure 7a). In osteoblast cultures derived from EAT of patients without coronary artery involvement, calcium was distributed unevenly in “nodules,” and areas without calcium were observed (Figure 7b). Colorimetric measurement of Alizarin Red concentration bound to calcium showed that osteoblasts from EAT of CAD patients produced 17% 25% more mineralized organic matrix than osteoblasts from the comparison culture (Figure 7c). Moreover, coronary artery calcium scores significantly correlated with the intensity of extracellular matrix mineralization in EAT-MSC cultures from CAD patients (*p* < 0.001; r = 0.68).

## 4. Discussion

The aim of this study was to evaluate and compare the immunophenotype, proliferative activity, and osteogenic potential of EAT-MSCs from patients with CAD and AS in order to elucidate the role of these cells in the pathogenesis of CAC.

Currently, coronary artery calcification in atherosclerosis is commonly attributed to the transdifferentiation of vascular smooth muscle cells (VSMCs) into osteoblast-like cells [11,12]. However, our study suggests that osteogenic differentiation of EAT-MSCs, rather than VSMC transdifferentiation, may represent a key mechanism in the pathogenesis of vascular calcification. This is based on the fact that MSCs are immature cells capable of osteogenic differentiation, whereas VSMCs are terminally differentiated cells with limited potential for phenotypic switching toward an osteoblast-like phenotype. In support of this, R. Alves et al. demonstrated that upon induction of osteogenesis, bone marrow MSCs expressed osteogenic markers and synthesized extracellular matrix more rapidly and efficiently compared to VSMCs [13].

The primary initiating event in the pathogenesis of CAC is atherosclerotic damage to the coronary vessels. The inflammatory process at the site of vascular injury creates a stress niche rich in pro-inflammatory cytokines (IFN-α, TNF-α, IL-1, IL-6), chemokines, and damage-associated molecular patterns. This niche contains activated immune cells, free radicals, and oxidized lipoproteins [14,15]. It is plausible that the combination of these pathological factors modifies the EAT-MSC niche, promoting their transformation into pathological calcifying vascular cells located in close proximity to the coronary arteries [16].

Despite shared risk factors, the pathogenesis of aortic valve calcification (AVC) in AS differs from that of CAC. This is supported by the prospective MESA cohort study, which included 6814 participants and revealed no association between AVC and CAC when comparing polygenic risk scores [17]. In our study, the clinical profiles of patients differed fundamentally: CAD patients presented with atherosclerotic coronary artery involvement and CAC, whereas AS patients had no such changes. Of particular note are the inflammatory markers. Although C-reactive protein levels were low (<2 mg/L) in both groups, CAD patients exhibited elevated IL-6 levels compared to AS patients. This pattern may indicate the presence of chronic inflammation, which is not always accompanied by a rise in CRP but may exert a profound influence on cell populations, including EAT-MSCs. IL-6 is a key mediator associated with both atherogenesis and osteogenic differentiation, making it a potentially significant factor in the acquisition of an osteogenic phenotype by MSCs [18,19]. CAD patients also had a higher prevalence of smoking. Smoking is a potent inducer of endothelial dysfunction, chronic inflammation, and oxidative stress. It has been demonstrated that tobacco smoke activates the osteogenic activity of MSCs. For instance, E. Wahl et al. showed that exposure of subcutaneous adipose tissue MSCs to 1% tobacco smoke during osteogenic induction enhanced the expression of osteogenic genes, including BGLAP, RUNX2, and SPP1 [20].

AS patients represent an optimal comparison group for our study, as coronary angiography revealed no coronary artery involvement in these patients. Furthermore, cardiovascular risk factors such as smoking and positive family history were less prevalent in the AS group. The main pathogenetic factor in AVC is increased mechanical load imposed by blood flow during each cardiac cycle [21]. In the most vulnerable areas of the valve, endothelial injury occurs, accompanied by infiltration of lipids and inflammatory cells that release reactive oxygen species (ROS) [22]. These factors promote the activation of an osteogenic phenotype in fibroblast-like valvular interstitial cells, which secrete a bone-like extracellular matrix [23].

We found that the proportion of CD73-positive MSCs in EAT cultures from CAD patients was significantly lower than in MSC cultures from AS patients. It is well established that MSCs are heterogeneous with respect to CD73 expression. K. Tan et al. demonstrated that MSCs derived from murine pericardial adipose tissue comprise two subpopulations with distinct CD73 expression levels: CD73high and CD73low [24]. Moreover, the level of CD73 expression may significantly affect stem cell properties, such as proliferation and differentiation capacity. For example, murine bone marrow MSCs with high CD73 expression have been shown to form colonies and proliferate more efficiently than CD73-negative cells [25]. Furthermore, CD73 overexpression in cancer stem cells accelerates proliferation and enhances the expression of stem cell markers [26].

In the present study, no significant differences in proliferative activity were observed between EAT-MSCs from CAD patients (with low CD73 expression) and those from AS patients (with high CD73 expression). This suggests that CD73 expression level does not substantially affect the proliferative capacity of cells from this anatomical location. This is consistent with the findings of K. Tan et al., who demonstrated that MSCs isolated from pericardial adipose tissue exhibit heterogeneity in CD73 expression while maintaining comparable proliferative potential [24].

Reduced expression of surface antigens is often associated with cellular senescence [27]. To exclude the influence of in vitro conditions, we assessed the accumulation of senescence biomarkers. At passage 3, MSC cultures from both groups contained a small proportion of senescent cells (4–6%). This confirms that reduced CD73 expression is a characteristic feature of EAT-MSC cultures from CAD patients rather than an artifact of culture conditions.

Reduced CD73 expression on MSCs is frequently associated with diminished differentiation potential. For instance, M. Takedachi et al. demonstrated that CD73 knockdown in murine bone marrow MSCs led to osteopenia with significantly reduced synthesis of osteoblastic markers [28]. However, in our study, MSC cultures from CAD patients, which contained approximately twofold fewer CD73-positive cells compared to AS-derived cultures, exhibited greater mineralization of the extracellular matrix. This likely reflects the loss of CD73-mediated anti-calcific protection, which is related to the enzyme’s function of converting extracellular adenosine monophosphate (AMP) to adenosine and inorganic phosphate. Deficiency of CD73, caused by mutations in the NT5E gene (encoding CD73), results in a marked decrease in extracellular adenosine, an anti-inflammatory and anti-calcifying molecule. Adenosine deficiency leads to increased activity of tissue-nonspecific alkaline phosphatase (TNAP), which degrades pyrophosphate—a major endogenous inhibitor of ectopic tissue calcification. Depletion of pyrophosphate removes the biochemical “brake” on mineralization, promoting calcium phosphate deposition and calcification in arterial walls [29].

The osteogenic potential of EAT-MSCs was assessed by the expression of key osteogenic genes, their corresponding proteins, and the intensity of extracellular matrix mineralization after differentiation. We found that following osteogenic induction, cells from CAD patients with CAC exhibited significantly higher expression of several osteogenic genes than MSCs from AS patients. For example, the transcription factor RUNX2, which initiates the cascade of multipotent cell differentiation into osteoblasts, was more highly expressed in MSCs from patients with coronary calcification than in cells from AS patients. RUNX2 is the master regulator of osteogenic differentiation and osteoblast formation. Therefore, the initial level of RUNX2 expression is critical for determining the osteogenic potential of MSCs [30,31]. mRNA levels of this gene were highest at early stages of differentiation, whereas protein levels were low. By the end of differentiation, RUNX2 protein levels had increased significantly, while its mRNA expression declined. This pattern suggests that RUNX2 expression in EAT-MSCs is regulated by negative feedback, consistent with the literature [32].

The SPP1 gene, encoding osteopontin, was expressed at later stages of osteoblast formation (by day 15), corresponding to the stage of extracellular matrix maturation [33]. Osteopontin serves as a structural protein providing a surface for cell attachment via integrin signaling, and also regulates the formation and growth of hydroxyapatite crystals in the extracellular matrix. At later stages of maturation, it acts as an inhibitor of mineral deposition, preventing excessive calcification [34]. We observed that SPP1 expression in EAT-MSCs from CAD patients was significantly higher than in cells from AS patients. Moreover, osteopontin levels in supernatants from CAD patients remained consistently higher than in AS-derived MSCs throughout the differentiation period, indicating active extracellular matrix formation. Blocking osteopontin function has been shown to inhibit osteogenic differentiation and promote adipogenesis [35]. Re-expression of osteopontin restores the balance between adipogenesis and osteogenesis in MSCs. Thus, increased expression of this protein promotes osteoblastogenesis, which is consistent with our findings.

Another marker indicating the commitment of EAT-MSCs from CAD patients toward osteoblast differentiation is alkaline phosphatase. Both ALPL mRNA levels and ALP protein concentrations in culture supernatants were significantly elevated compared to AS-derived MSC cultures. Alkaline phosphatase is a key enzyme in hydroxyapatite crystal formation, acting by increasing local inorganic phosphate levels. Our data are in agreement with the literature. For example, Yun Hee Kim et al. showed that bone marrow MSCs with low ALP expression retained multipotency, whereas cells with high ALP expression were committed to the osteoblast lineage [36].

Visualization of the extracellular matrix by Alizarin Red staining confirmed the greater intensity of osteogenesis in EAT-MSC cultures from CAD patients compared to AS-derived cells. The staining intensity of the extracellular matrix correlated with the coronary calcium scores of patients with atherosclerotic coronary artery disease, providing further evidence for the involvement of EAT-MSCs in CAC formation. In conclusion, MSCs derived from CAD patients with CAC demonstrated significantly higher osteogenic potential compared to EAT-MSCs from AS patients.

## 5. Conclusions

Thus, the combination of cardiovascular risk factors and pre-existing coronary artery damage in CAD patients may contribute to the “reprogramming” of EAT-MSCs under these pathological conditions, which is manifested by their commitment to osteogenic differentiation in vitro. The identified correlation between the degree of CAC and the intensity of extracellular matrix mineralization by EAT-MSCs suggests that these cells are involved in the pathogenesis of cardiac vascular calcification.

## Figures and Tables

**Table 1:** Clinical and anamnestic characteristics of the study groups.

Parameter	CAD Group (n = 30)	AS Group (n = 30)	*p*
Male, n (%)	21 (70)	19 (63)	0.053
Age, years, Me (Q25; Q75)	67 (58; 72)	66 (53; 69)	0.061
Body mass index, kg/m^2^, Me (Q25; Q75)	28.5 (25.3; 31.1)	27.3 (24.5; 30.8)	0.06
Arterial hypertension, n (%)	23 (76)	14 (46)	0.042
Dyslipidemia, n (%)	7 (23)	4 (13.3)	0.037
TC, mmol/L, Me (Q25; Q75)	3.6 (3.2; 3.9)	3.55 (3.4; 3.9)	0.192
HDL-C, mmol/L, Me (Q25; Q75)	1.7 (0.9; 1.2)	1.05 (0.9; 1.1)	0.827
LDL-C, mmol/L, Me (Q25; Q75)	1.8 (1.7; 2.2)	1.7 (1.6; 2.02)	0.726
VLDL-C, mmol/L, Me (Q25; Q75)	0.5 (0.4; 0.55)	0.53 (0.5; 0.72)	0.524
TAG, mmol/L, Me (Q25; Q75)	1.2 (1.03; 1.5)	1.18 (1.11; 1.4)	0.823
Lp(a), nmol/L, Me (Q25; Q75)	46 (42; 60)	90 (69; 95)	0.018
Smoking, n (%)	12 (40)	4 (13.3)	0.027
Family history of CAD, n (%)	6 (20)	1 (3.3)	0.032
Previous myocardial infarction, n (%)	4 (13.3)	0 (0)	0.011
Previous stroke/TIA, n (%)	1 (3.3)	1 (3.3)	0.046
History of CHF, n (%)	7 (23)	12 (40)	0.041
CHF class I, n (%)	1 (3.3)	1 (3.3)	0.057
CHF class II, n (%)	6 (20)	4 (13.3)	0.049
CHF class III, n (%)	1 (3.3)	4 (13.3)	0.025
CHF class IV, n (%)	0 (0)	0 (0)	-
Atherosclerosis of other vascular beds, n (%)	9 (30)	1 (3.3)	0.042
Single-vessel coronary disease, n (%)	12 (40)	0 (0)	-
Two-vessel coronary disease, n (%)	7 (23)	0 (0)	-
Multivessel coronary disease, n (%)	12 (40)	0 (0)	-
SYNTAX Score (<22 points), n (%)	6 (20)	0 (0)	–
SYNTAX Score (23–31 points), n (%)	10 (33.3)	0 (0)	–
SYNTAX Score (≥32 points), n (%)	14 (46.7)	0 (0)	–
CRP, mg/L, Me (Q25; Q75)	1.7 (0.5; 1.9)	1.9 (0.3; 2.3)	0.48
IL-6, pg/mL, Me (Q25; Q75)	24.8 (9.6; 36.4)	8.2 (3.5; 16.2)	0.001
Coronary calcium score, Me (Q25; Q75)	1396 (1295; 1893)	0	0.001

Note: data are presented as median (Me) with 25th and 75th percentiles (Q25; Q75) or as number (percentage). CAD—coronary artery disease; AS—aortic stenosis; TIA—transient ischemic attack; CHF—chronic heart failure; TC—total cholesterol; HDL-C—high-density lipoprotein cholesterol; LDL-C—low-density lipoprotein cholesterol; VLDL-C—very low-density lipoprotein cholesterol; TAG—triacylglycerols; Lp(a)—lipoprotein(a); CRP—C-reactive protein; IL-6—interleukin-6.

**Table 2:** Primers used in real-time RT-PCR.

Gene Name	Primer	
	Forward	Reverse
Genes used for normalization in expression level calculations		
*GAPDH*	AGCCACATCGCTCAGACAC	GCCCAATACGACCAAATCC
*B2M*	TCCATCCGACATTGAAGTTG	CGGCAGGCATACTCATCTT
*ACTB*	CATCGAGCACGGCATCGTCA	TAGCACAGCCTGGACAGCAAC
Genes of Interest		
*RUNX2*	AGATGGACCTCGGGAACCCA	TGAGGCGGGACACCTACTCT
*SP7*	TGCTTGAGGAGGAAGTTCAC	AGGTCACTGCCCACAGAGTA
*BGLAP*	TCACACTCCTCGCCCTATTG	TAGCGCCTGGGTCTCTTCAC
*ALPL*	AGGGTCAGCTCCACCACAAC	GCCTTCACCCCACACAGGTA
*SPP1*	CATCACCTGTGCCATACCAGTT	TTGGAAGGGTCTGTGGGGCTA

**Table 3:** Proportion of MSCs expressing major surface markers in cultures from CAD and AS patients at passage 3.

Surface Marker	MSCs from CAD Patients (n = 30)	MSCs from AS Patients (n = 30)	*p*
CD105, %, Me (Q25; Q75)	90.38 (84.29; 99.3)	99.47 (86.39; 100)	0.043
CD73, %, Me (Q25; Q75)	49.2 (34.3; 69.1)	73.05 (65.9; 89.2)	0.0012
CD90, %, Me (Q25; Q75)	92.9 (82.26; 100)	94.6 (89.42; 100)	0.68
CD45, %, Me (Q25; Q75)	0.09 (0.01; 0.15)	0.12 (0.05; 0.16)	0.06
CD34, %, Me (Q25; Q75)	0.14 (0.05; 0.18)	0.11 (0.03; 0.16)	0.25
CD31, %, Me (Q25; Q75)	0.5 (0.2; 0.9)	1.2 (0.6; 1.9)	0.012
HLA-DR, %, Me (Q25; Q75)	0 (0; 0)	0 (0; 0)	–

Note: MSCs—mesenchymal stem cells; CAD—coronary artery disease; AS—aortic stenosis.

## Data Availability

The data presented in this study are available on request from the corresponding author due to ethical reasons.

## Abbreviations

The following abbreviations are used in this manuscript:

MSCs	Mesenchymal stem cells
EAT	Epicardial adipose tissue
CAC	Coronary artery calcification
CAD	Coronary artery disease
AS	Aortic stenosis
VSMCs	Vascular smooth muscle cells
AVC	Aortic Valve Calcification
